# Earlier surveillance colonoscopy programme improves survival in patients with ulcerative colitis associated colorectal cancer: results of a 23-year surveillance programme in the Japanese population

**DOI:** 10.1038/sj.bjc.6601247

**Published:** 2003-09-30

**Authors:** K Hata, T Watanabe, S Kazama, K Suzuki, M Shinozaki, T Yokoyama, K Matsuda, T Muto, H Nagawa

**Affiliations:** 1 Department of Surgical Oncology, The University of Tokyo, 7-3-1 Hongo, Bunkyo-ku, Tokyo 113-8655, Japan; 2 Department of Surgery, The Cancer Institute Hospital, 1-37-1 Kami-Ikebukuro Toshima-ku, Tokyo 170-8455, Japan

**Keywords:** colitic cancer, ulcerative colitis, surveillance colonoscopy, dysplasia, colorectal cancer

## Abstract

Patients with long-standing ulcerative colitis (UC) are known to have an increased risk for the development of colorectal cancer (CRC). The aim of this study was to clarify the cumulative risk for the development of dysplasia or invasive cancer and the effectiveness of surveillance colonoscopy in the Japanese population. A total of 217 patients received a total of 1027 surveillance colonoscopies between January 1979 and December 2001 at the University of Tokyo hospital. Patients with invasive cancer found in the surveillance group were compared to those referred to our hospital from the other hospitals without surveillance colonoscopy. Surveillance colonoscopy confirmed 15 patients with definite dysplasia. Of these, five were proved to have invasive cancer in the resected specimens. The cumulative risk for the development of invasive cancer at 10, 20, and 30 years was 0.5, 4.1, and 6.1%, respectively, while that for the development of definite dysplasia at 10, 20, and 30 years was 3.1, 10.0, and 15.6%, respectively. All the patients with invasive cancer in the surveillance group remained alive, while three out of four patients in the nonsurveillance group died. Our surveillance programme is useful for detecting UC-associated CRC, and survival may be improved by surveillance colonoscopy.

Patients with long-standing ulcerative colitis (UC) are known to have an increased risk for the development of colorectal cancer (CRC) (Ekbom *et al*, 1990) The recent meta-analysis study reported that the cumulative risk of developing CRC is estimated as 2% by 10 years, 8% by 20 years, and 18% by 30 years in patients with UC (Eaden *et al*, 2001). Ulcerative colitis-associated CRC is different from sporadic CRC in several ways. Patients with UC-associated CRC are younger than those with sporadic CRC, and even children are at risk for CRC in UC patients (Eaden *et al*, 2001). In addition, UC-associated CRC tends to be widespread and is difficult to detect by colonoscopy (Morson and Pang, 1967). Therefore, a specific surveillance programme should be established for patients with long-standing UC. The well-known risk factors for UC-associated CRC are the duration and extent of disease (Gyde *et al*, 1988; Ekbom *et al*, 1990; Leidenius *et al*, 1991). It is generally accepted that patients with total colitis for 8 years or longer and those with left-sided colitis for 12–15 years or longer should receive surveillance colonoscopy every 1 or 2 years in the Western countries (Winawer *et al*, 1997; Farrell and Peppercorn, 2002) However, the effectiveness of such programmes is still controversial (Lynch *et al*, 1993; Axon, 1994). In addition, the cumulative risk for the development of dysplasia or cancer has not been reported in the Asian populations. The aim of this study was to clarify the cumulative risk for the development of dysplasia or cancer and the effectiveness of surveillance colonoscopy for the detection of UC-associated CRC in the Japanese population. Our report outlines the results of a 23-year surveillance colonoscopy programme for detecting CRC in long-standing UC in the Japanese population, and as such, represents the first report of its kind in Japan.

## MATERIAL AND METHODS

### Patients' selection

We performed a surveillance colonoscopy programme at the University of Tokyo hospital over a period of 23 years between January 1979 and December 2001. Surveillance colonoscopy was performed annually from 7 years after the onset of symptoms for patients suffering from total UC (proximal to the splenic flexure) and left-sided UC (distal to the splenic flexure). In several patients, surveillance colonoscopy scheduled for 7 years from the onset was performed several months earlier than 7 years. Such patients were included in this study. The extent of disease was defined with macroscopic findings at colonoscopy. Patients with proctitis were excluded from this study. Surveillance colonoscopy was also performed for those who had undergone subtotal colectomy and ileo-rectal anastomosis (IRA) to survey the retained rectum. Patients with known dysplasia or cancer at the time of referral were excluded. Those without evidence of dysplasia at the time of referral were entered into this programme.

The extent of disease was classified at the first surveillance colonoscopy. If the observed extent was less extensive than what had been observed previously, the greatest extent of disease was used for classification of the extent. If the observed extent had increased, it was defined as disease progression. A total of 217 patients were retrospectively reviewed in this study (surveillance group). In all, 123 patients with total UC (one patient underwent IRA during surveillance because of intractable disease), 68 with left-sided UC (in 14 of whom the disease had progressed to total colitis) and 27 with post-IRA were retrospectively reviewed. A total of 1027 colonoscopies were performed for the purpose of surveillance. During the same period, four patients, who had not received surveillance colonoscopy and who had been diagnosed at other hospitals as having symptomatic invasive cancer, were referred to us for surgical treatment (nonsurveillance group).

### Surveillance colonoscopy

We offered patients annual total colonoscopy, preferentially during the remission state. Biopsy specimens were taken from flat mucosa at least every 10 cm of the whole colorectum, and additional biopsy specimens were taken from lesions with any remarkable endoscopic abnormalities such as those that were elevated and those with colour changes.

### Histopathology

Biopsy specimens were fixed with formalin and stained with Haematoxylin and eosin, and graded as high-grade dysplasia (HGD), low-grade dysplasia (LGD), indefinite for dysplasia (IND), or negative for dysplasia according to the criteria of the Inflammatory Bowel Disease/Dysplasia morphology study group (Riddell *et al*, 1983).

### Follow-up

Total proctocolectomy was performed when patients were found to have HGD. We performed follow-up colonoscopy within 3 months for patients with LGD or IND. Surgery was performed for patients with persistent LGD or LGD with dysplasia-associated lesion or mass. A well-defined elevated lesion resembling a sporadic adenomatous polyp without dysplastic change of the surrounding mucosa was treated as coincidental adenoma and polypectomy was performed. Such lesions were not included in the category of dysplasia in this study. Otherwise, annual colonoscopy was performed.

### Evaluation

The cumulative risk for dysplasia and invasive cancer in the surveillance group was evaluated. Patients with invasive cancer in both surveillance and nonsurveillance groups were reviewed in terms of the age of onset, gender, duration of disease, Dukes' classification, and survival.

### Statistics

StatView software (SAS Institute Inc, Cary, NC, USA) was used for statistical analyses. The cumulative dysplasia-free and cancer-free rates were calculated by the Kaplan–Meier method.

## RESULTS

### Results of surveillance colonoscopy

**Figure 1:**
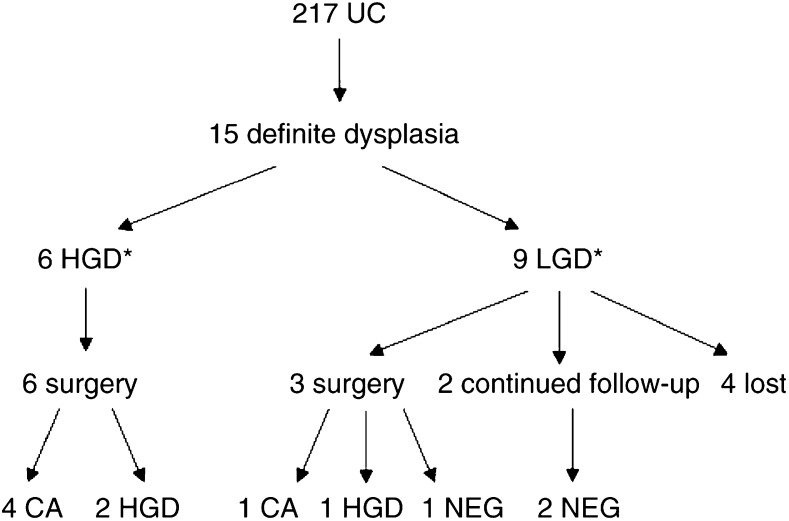
Summary of the results of a surveillance colonoscopy programme in the Japanese population (^*^the highest degree of dysplasia found in colonoscopy was used. CA=invasive cancer; HGD=high-grade dysplasia; LGD=low-grade dysplasia ; NEG=negative for dysplasia).

High-grade dysplasia or LGD were detected in 15 patients through our surveillance programme. Of these, twelve patients suffered from total colitis. Two patients were status post-IRA, both of whom had had total colitis preoperatively. Only one patient had left-sided colitis. A summary of the results of the surveillance colonoscopy is shown in Figures 1

and 2

**Figure 2:**
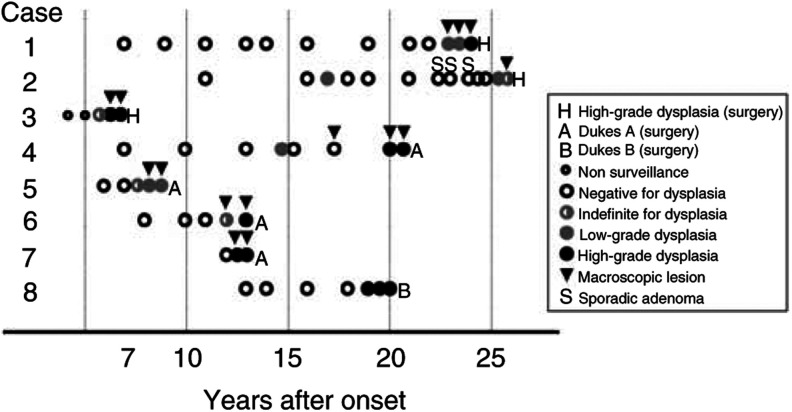
Results of surveillance colonoscopies in patients who were found to have high-grade dysplasia or invasive carcinoma in resected specimen.

.

Six patients were found to have HGD at surveillance colonoscopy, all of whom underwent colectomy. Four of them had invasive cancer and two had HGD in the resected specimens. Nine patients were found to have LGD at surveillance colonoscopy. Of the three patients who underwent colectomy, one had invasive cancer and one had HGD. The other patient was found to have flat LGD at surveillance colonoscopy, and negative for dysplasia at the two following colonoscopies. However, she preferred to undergo colectomy to close follow-up colonoscopy, and no dysplasia was found in the resected specimens. Two patients continued to receive surveillance colonoscopy and no dysplasia was found after LGD was detected. Four patients were lost to follow-up. Two of them had undergone operation in other hospitals, and pathological reports were not available for review.

### Dysplasia-free survival

**Figure 3:**
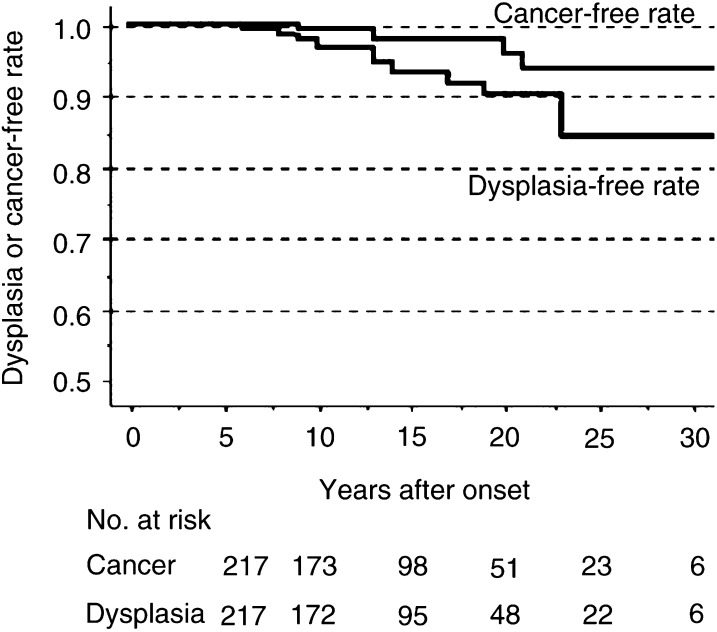
The cumulative definite dysplasia- and cancer-free survival rates in patients with long-standing ulcerative colitis in the Japanese population.

The cumulative dysplasia (HGD or LGD)-free survival curve and cumulative cancer-free survival curve in patients with long-standing UC in the surveillance group are shown in Figure 3

. The cumulative risk for the development of invasive cancer at 10, 20, and 30 years (95% confidence interval) was 0.5% (0–1.5), 4.1% (0–8.3), and 6.1% (0.2–12.0), respectively, while that for the development of definite dysplasia at 10, 20, and 30 years (95% confidence interval) was 3.1% (0.6–5.6), 10.0% (4.3–15.7), and 15.6% (6.4–24.8), respectively.

### Prognosis, surveillance group *vs* nonsurveillance group

The patients with invasive cancer in the surveillance group had better prognoses than those in the nonsurveillance group. The clinicopathological features in both groups are listed in Table 1

**Table 1:** Clinicopathological features of the patients with ulcerative colitis-associated colorecal cancer

**Case**	**Age of Onset (years)**	**Age at operation (years)**	**Duration (years)**	**Histology^a^**	**Depth**	**Dukes**	**Location^b^**	**Extent^c^**	**Survival**
*Surveillance group*									
4	16	37	21	mod,por⩾well	sm	A	R	L	Alive 7y5m
5	56	65	9	well to mod	sm	A	S	T	Alive 5y10m
6	28	41	13	well	sm	A	R	T(IRA)	Alive 16y10m
7	36	49	13	well	mp	A	C	T	Alive 12y10m
8	21	41	20	well>mod>por	ss	B	R	T	Alive 9y8m
									
*Non surveillance group*									
	23	44	21	well	ss	B	D	T	Alive 11y1m
	29	42	13	sig	se	C	S	T	Dead 1y
	20	40	20	muc>mod>por	se	C	D	T	Dead 2y5m
	29	47	18	sig	se	C	D	T	Dead 1m

y=years; m=months.

a muc=mucinous adenocarcinoma; well=well differentiated adenocarcinoma; mod=moderately differentiated adenocarcinoma; por=poorly differentiated adenocarcinoma; sig=signet-ring cell carcinoma.

b R=rectum; S=sigmoid colon; D-descending colon; C=caecum.

c T=total colitis; L=left-sided colitis.

. Five patients in the surveillance group were found to have invasive cancer. Dukes' stages in the surveillance group were A in four and B in one, whereas those in the nonsurveillance group were C in three, and B in one. All the patients with invasive cancer in the surveillance group remained alive, whereas three out of four patients died. The mean postoperative follow-up period was 126 months (70–202), and 41 months (1–133) for the surveillance and nonsurveillance group, respectively.

## DISCUSSION

We performed a surveillance programme to detect UC-associated CRC for 23 years, which we believe to be the first surveillance programme reported in the Asian population. Our data suggest that our surveillance programme was useful for detecting UC-associated CRC, although the study was not performed in a randomised manner and lead-time bias together with selection bias could not be avoided. Some authors doubt whether surveillance colonoscopy can detect UC-associated CRC earlier and thereby improve the prognosis (Axon, 1994). However, it is generally accepted that surveillance colonoscopy is important and is the only way for detecting UC-associated CRC at this time (Winawer *et al*, 1995). Therefore, this kind of study could not be performed in a randomised manner.

Our programme successfully identified UC-associated CRC at an earlier stage than other surveillance studies. Dukes' stages in the surveillance group were A in four and B in one, whereas those in the nonsurveillance group were C in three patients and B in one patient. It had been reported that patents with UC-associated CRC at Dukes' A and B showed a good survival rate, but those at Dukes' C showed an extremely poor prognosis (Heimann *et al*, 1992). Our data are compatible with that report. Patients with UC-associated CRC in the surveillance group showed better prognoses than those in the nonsurveillance group. All the patients in the surveillance group remained alive, whereas three patients out of four in the nonsurveillance group died. The duration of UC was approximately 5 years shorter in the surveillance group, and this could result in lead-time bias. However, the follow-up period was considered to be long enough to evaluate the prognoses. Since all the patients basically underwent surveillance colonoscopy at the time of referral or 7th year from the onset, it was difficult to select an appropriate control group. In our series, several patients refused further surveillance colonoscopy, but no such patients have developed invasive cancer so far. Four patients, who did not receive surveillance colonoscopy and were diagnosed at other hospitals as having symptomatic invasive cancer, were referred to us for the surgical treatment. These four were selected as the control.

The cumulative risk of UC-associated CRC was calculated in the surveillance group. There had been criticism that most of the previous reports had statistical problems in calculating the cumulative risk (Collins *et al*, 1987). In our series, patients with UC-associated CRC or dysplasia referred from the other hospitals were excluded to eliminate the bias. We believe this report to be the first report in the Asian population. The cumulative risk for invasive cancer in our series seems lower than those reported in most of the Western countries (Biasco *et al*, 1995; Lindberg *et al*, 1996), but may be higher than those reported in Denmark, Czechoslovakia, and Israel (Nedbal and Maratka, 1968; Gilat *et al*, 1974; Hendriksen *et al*, 1985). The corresponding figure for dysplasia or cancer in our series is almost identical to that reported in a very large series in the UK (Lennard-Jones *et al*, 1990), and that for invasive cancer calculated by meta-analysis (Eaden *et al*, 2001). These results suggest that UC-associated cancer in the Japanese population may be lower than that in most of the American and European populations, but higher than that in Denmark, Czechoslovakia, and Israel, although the confidence interval was wide in our series.

In our series, patients with total UC tended to have a higher risk for developing HGD or invasive cancer than those with left-sided colitis, but the difference was not statistically significant. Disease extent is a confusing issue, since the definition is inconsistent. We defined total colitis as colitis extending proximal to the splenic flexure, and left-sided colitis as distal to it by macroscopic findings at colonoscopy. We included proctosigmoiditis, but not proctitis in the category of left-sided colitis.

Disease progression is another important issue. Previous studies reported that a considerable number of patients with left-sided UC experienced disease progression (Leijonmarck *et al*, 1990; Farmer *et al*, 1993). In our series, 25 patients out of 137 (18%) with total colitis were first regarded as being of the less extensive type. The disease progressed from left-sided to total colitis after 7 years from the onset in 14 of these patients. None of them has been found to have dysplasia, so far. However, dysplasia or cancer would be missed if left-sided colitis were excluded from the surveillance programme. Connell *et al* (1994) also pointed out this ‘presumed left-sided’ problem. In addition, it has been reported that the cancer risk and the disease duration before the diagnosis of cancer were the same in both total and left-sided colitis (Nugent *et al*, 1991; Sugita *et al*, 1991). Therefore, left-sided colitis should not be totally excluded from the surveillance programme. However, the optimum interval and starting point of surveillance for patients with left-sided colitis should wait for cost-effectiveness studies of the surveillance programme.

Our cases suggest that the starting point of surveillance colonoscopy should be earlier than in previous reports. The starting point in our series is 7 years. The guidelines of the World Health Organization recommend that surveillance colonoscopy for total colitis (extending at least to the hepatic flexure) should be started from 8 years after the onset of symptoms, and that for left-sided colitis (more distal involvement) it should be performed from 12 to 15 years (Winawer *et al*, 1995). Other guidelines from the American Journal of Gastroenterology recommend that surveillance colonoscopy for both total and left-sided colitis should be started from 8 to 10 years (Kornbluth and Sachar, 1997). Bernstein *et al* reported that 12% of the patients with long-standing total colitis were found to have cancer or dysplasia at the first surveillance colonoscopy in 10 surveillance programmes, and concluded that dysplasia surveillance should be performed earlier (Bernstein *et al*, 1994). In our series, two patients were found to have dysplasia by colonoscopy within 7 years of onset. One patient developed LGD 5 years and 8 months after onset (this patient was not included in this study). Furthermore, serosal invasion was found in the surgical specimen after an 8-month interval of refusing colectomy against medical advice. In this case, refractory disease and persistent anaemia made us promote surveillance colonoscopy before 7 years after onset. In another patient, surveillance colonoscopy that was performed several months before reaching that point, but set for 7 years after the onset revealed dysplasia, and the surgical specimen revealed HGD. Surveillance colonoscopy starting from 7 years after onset might have rescued the patient in the latter case, but not in the former case. However, the cost would increase, if we started surveillance colonoscopy from 5 years after onset.

The optimum frequency of surveillance colonoscopy is controversial, but many reports adopted a 1-or-2-year interval. Annual colonoscopy will double the cost but may increase sensitivity as compared to the biannual colonoscopy. Moreover, we have to take it into consideration that UC-associated CRC may advance faster than sporadic CRC. The answer to this question should wait for the cost–benefit analysis.

Some authors criticised poor compliance of the surveillance colonoscopy. In our series, 109 patients continued receiving surveillance colonoscopy, 20 underwent colectomy, 19 moved to the other hospitals, 59 were lost to follow-up with reasons unknown, nine refused colonoscopy, and one died of congestive heart failure. In all, 30% of the patients failed to adhere to our programme due to refusal or loss to follow-up. Compliance in our series was not as good as that in the St Marks' series (Connell *et al*, 1994). Further efforts need to be made to maintain good compliance.

## CONCLUSIONS

In what we believe to be the first series in the Asian population, we performed a surveillance colonoscopy programme for long-standing UC for 23 years, starting such surveillance earlier than those reported previously, which successfully detect dysplasia or cancer at an earlier stage.
